# Non-Invasive Ventilation Support during Hospitalization for SARS-CoV-2 and the Risk of Venous Thromboembolism

**DOI:** 10.3390/jcm13102737

**Published:** 2024-05-07

**Authors:** Carmine Siniscalchi, Andrea Ticinesi, Antonio Nouvenne, Angela Guerra, Alberto Parise, Lorenzo Finardi, Nicoletta Cerundolo, Beatrice Prati, Loredana Guida, Tiziana Meschi

**Affiliations:** 1 General and Specialistic Medicine Department, Azienda Ospedaliero-Universitaria di Parma, 43126 Parma, Italy; 2 Department of Medicine and Surgery, University of Parma, Via Antonio Gramsci 14, 43126 Parma, Italy; andrea.ticinesi@unipr.it (A.T.); antonio.nouvenne@unipr.it (A.N.); angela.guerra@unipr.it (A.G.); aparise@ao.pr.it (A.P.); lfinardi@ao.pr.it (L.F.); ncerundolo@ao.pr.it (N.C.); bprati@ao.pr.it (B.P.); lguida2@ao.pr.it (L.G.); tiziana.meschi@unipr.it (T.M.)

**Keywords:** SARS-CoV-2, venous thromboembolism, non-invasive ventilation support

## Abstract

**Background/Objectives:** Although SARS-CoV-2 infection is a significant risk factor for venous thromboembolism (VTE), data on the impact of the use of non-invasive ventilation support (NIVS) to mitigate the risk of VTE during hospitalization are scarce. **Methods:** Data for 1471 SARS-CoV-2 patients, hospitalized in a single hub during the first pandemic wave, were collected from clinical records, including symptom duration and type, information on lung abnormalities on chest computed tomography (CT), laboratory parameters and the use of NIVS. Determining VTE occurrence during hospital stays was the main endpoint. **Results:** Patients with VTE (1.8%) had an increased prevalence of obesity (26% vs. 11%), diabetes (41% vs. 21%), higher CHA2DS2VASC score (4, IQR 2–5 vs. 3, IQR 1–4, age- and sex-adjusted, *p* = 0.021) and cough (65% vs. 44%) and experienced significantly higher rates of NIVS (44% vs. 8%). Using a stepwise multivariate logistic regression model, the prevalence of electrocardiogram abnormalities (odds ratio (OR) 2.722, 95% confidence interval (CI) 1.039–7.133, *p* = 0.042), cough (OR 3.019, 95% CI 1.265–7.202, *p* = 0.013), CHA2DS2-VASC score > 3 (OR 3.404, 95% CI 1.362–8.513, *p* = 0.009) and the use of NIVS (OR 15.530, 95% CI 6.244–38.627, *p* < 0.001) were independently associated with a risk of VTE during hospitalization. NIVS remained an independent risk factor for VTE even after adjustment for the period of admission within the pandemic wave. **Conclusions:** Our study suggests that NIVS is a risk factor for VTE during hospitalization in SARS-CoV-2 patients. Future studies should assess the optimal prophylactic strategy against VTE in patients with a SARS-CoV-2 infection candidate to non-invasive ventilatory support.

## 1. Introduction

Venous thromboembolism (VTE), which includes deep vein thrombosis (DVT) and pulmonary embolism (PE), is a worldwide health problem. The annual incidence of VTE in the general population is estimated to be around 1–2 cases per 1000 persons [1,2]. It is estimated that PE, the most feared presentation of VTE, causes 6–12 deaths per 100,000 people, corresponding to around 40,000 deaths per year in the European Region [3,4]. Hospitalized patients with acute medical illness are at high risk of VTE during hospitalization. Several hospitalization-specific factors (such as immobilization, sedation, vasopressors or central venous catheters) together with individual patient-related factors (such as age, cancer, obesity, immobilization, history of personal or familiarities for VTE, sepsis, respiratory or heart failure, pregnancy, stroke, trauma or recent surgery) together contribute to this risk [5,6]. Thus, all hospitalized patients are assessed for VTE risk and are often administered thromboprophylaxis. SARS-CoV-2 is a viral respiratory tract infection caused by SARS-CoV-2 infection that led to a pandemic in early 2020 in Western countries after spreading from China. SARS-CoV-2 infection leads to endothelial damage, with vascular thrombosis and micro-angiopathy, and occlusion of alveolar capillaries with a significant intussusceptive angiogenesis and new vessel [7]. There are several biomarkers associated with the severity of COVID-19, including C-reactive protein (CRP), D-dimer levels and troponin [8]. COVID-19 patients who die exhibit higher levels of troponin I, D-dimer and CRP when compared with COVID-19 survivors [9]. Thus, prognostic levels of biomarkers associated with COVID-19 may assist in understanding the progression of the disease. Severe SARS-CoV-2 is often complicated with coagulopathy, with related prothrombotic effects and a high risk of VTE [5,10,11]. International societies strongly recommend the use of thromboprophylaxis in all hospitalized patients with SARS-CoV-2 infection [12,13,14,15]. Despite thromboprophylaxis administration, hospitalization of SARS-CoV-2 patients is frequently complicated by VTE. While the incidence of thrombotic complications in critical SARS-CoV-2 patients is very high, as the incidence in patients under non-invasive respiratory ventilation support (NIVS) is still unknown [16,17]. The specific incidence of thrombotic events in each of the clinical scenarios within the broad spectrum of severity of SARS-CoV-2 is not clearly established and this has not allowed the implementation of thromboprophylaxis or anticoagulation for routine care in SARS-CoV-2 patients [18,19]. In this context, the purpose of the present study was to investigate the incidence of VTE in patients hospitalized for suspected or confirmed SARS-CoV-2 infection during the first pandemic wave, especially in relation to the administration of NIVS for respiratory failure during hospitalization.

## 2. Materials and Methods

### 2.1. Patient Characteristics and Data Collection

This study was conducted in an Internal Medicine Unit of a large teaching hospital in Northern Italy (Parma University-Hospital), which was appointed as the main hub for the care of SARS-CoV-2 patients for the whole Parma province (approximately 450,000 inhabitants) in the earliest phases of the first wave [20]. Two groups of patients hospitalized with SARS-CoV-2 from 28 February 2020 to 10 June 2020 were retrospectively enrolled after applying the exclusion and inclusion criteria. We arbitrarily divided this time frame, corresponding to the first pandemic wave, into two periods named “first period” from 28 February 2020 to 22 March 2020, and “second period” from 22 March 2020 to 10 June 2020. These subclassifications were made to distinguish the first period characterized by a high rate of hospitalization/per day and exceptional burden for the whole healthcare system from the second period (corresponding to the descending phase of the same pandemic wave) characterized by a lower rate of hospitalization/per day.

Only patients aged ≥18 years old with SARS-CoV-2 infection confirmed by reverse transcriptase polymerase-chain reaction (RT-PCR) by nasopharyngeal swab performed upon urgent admission, or with chest computed tomography (CT) evidence of lung interstitial involvement with a high radiological and clinical suspicion of COVID-19 were included in the study. Thus, subjects with positive chest CTs, but negative RT-PCR tests collected via nasopharyngeal swabs performed upon hospital admission were included in the study and labelled “suspect COVID-19 cases”. In the earliest phases of the pandemic, the epidemiological context was characterized by a high daily number of hospital admissions for respiratory failure (up to 70 admissions per day), so the presence of a chest CT showing interstitial abnormalities allowed doctors to reasonably assume that patients had COVID-19 even if the first RT-PCR test was negative. Conversely, all subjects with missing data on variables needed for the study and subjects who were transferred to other wards (i.e., with missing data on the outcome) were excluded.

For each participant we collect the number and types of comorbidities (including dyslipidemia, hypertension, obesity, diabetes, cancer, heart diseases and chronic kidney disease), demographic data (sex and age), number of drugs prescribed, clinical presentation of SARS-CoV-2 (i.e., chest CT abnormalities, symptoms and their duration and vital signs) and the results of lab tests performed on admission, including blood cell count, arterial blood gas analysis, serum creatinine, predicted glomerular filtration rate, D-dimer, CRP and procalcitonin (PCT). We analyzed the following parameters: arterial blood gas analysis (RADIOMETER ABL instrument); blood test: WBC, 1000/mm^3^ (flow cytometry method, normal values 4.00–10.00); neutrophils, 1000/mm^3^ (flow cytometry method, normal values 1.80–8.00); lymphocytes, 1000/mm^3^ (flow cytometry method, normal values 1.00–4.00); creatinine, mg/dL (Jaffè method, normal values 0.5–1.4); C-reactive protein, mg/L (Atellica CH High Sensitivity C-Reactive Protein, normal values 0.16–10.00); PCT, ng/mL (chemiluminescence immunometric assay, normal values 0.00–0.50); D-dimer, ng/dL (immunological method, normal values < 500); PT and aPTT (coagulation method, normal values INR ratio 0.86–1.20; aPTT ratio 0.82–1.18); fibrinogen, mg/dL (CLAUSS method, 150–400) normal values: pH (7.36–7.44); HCO_3_^−^, mmol/L (22–26); pCO_2_, mmHg (38–42) and pO_2_, mmHg (85–100). By the use of chest CT visual score, the extension of pulmonary abnormalities and infiltrates was estimated through calculations detailed elsewhere [21]. We also collected data on treatments administered during hospital stay and outcome (survival vs. death) for all participants.

Ethics Committee approval was obtained (Comitato Etico dell’Area Vasta Emilia Nord, Emilia-Romagna region) under the ID 273/2020/OSS/AOUPR as part of a larger project on the characteristics of patients hospitalized with confirmed or suspected COVID-19 during the first pandemic wave. All participants, who were contactable by phone or for follow-up reasons, provided written informed consent for participation. For all other cases, the Ethics Committee, in accordance with the guidelines in force at the moment of approval, waived written informed-consent collection due to the retrospective design of the study.

### 2.2. Statistical Analyses

Variables were expressed as median and interquartile range (IQR) or percentages, as appropriate. The characteristics of participants were compared with the Mann–Whitney or chi-square tests, with adjustment for age and sex with Quade non-parametric ANCOVA (continuous variables) or binary logistic regression (dichotomous variables). The factors independently associated with VTE in both groups were investigated with stepwise multivariate logistic regression models considering the participants altogether and after partition according to pandemic wave. Diabetes, CHA2DS2-VASC SCORE, obesity, admission electrocardiogram (ECG) abnormalities, cough, dyspnea, neutrophil count, C-reactive protein, non-invasive ventilation support and CHA2DS2-VASC SCORE > 3, CHA2DS2-VASC SCORE > 4 were considered entries in these multivariate models. Additional analyses were also made after the categorization of participants of both periods by VTE status.

Analyses were performed with the SPSS statistical package (v. 28, IMB, Armonk, NY, USA), considering *p* values < 0.05 as statistically significant.

## 3. Results

### 3.1. General Characteristics of Population

We included in this study 1471 patients admitted from 28 February 2020 to 10 June 2020, of which 27 suffered from VTE and 1444 did not suffer from VTE. Their clinical characteristics are compared in Table 1. Patients without VTE have less comorbidities than those with VTE. The patients with DVT had an increased prevalence of obesity (median 26% vs. 11%), diabetes (41% vs. 21%) and a higher CHA2DS2VASC score than non-DVT SARS-CoV-2 patients (4, IQR 2–5 vs. 3 pt, IQR 1–4, age- and sex-adjusted, *p* = 0.021). SARS-CoV-2 patients with DVT took more antiepileptic drugs (19% vs. 7%) and insulin (19% vs. 7%) than non VTE patients. The clinical presentation was also different with the prevalence of a cough (65% vs. 44%) in VTE patients. Patients with DVT had a higher prevalence of ECG abnormalities (74% vs. 52%), white-blood-cell count (9.16, IQR 6.62–12.50 vs. 6.99 1000/mm^3^, IQR 5.12–9.55, age- and sex-adjusted, *p* = 0.011), neutrophil count (7.65, IQR 5.43–10.13 vs. 5.32 1000/mm^3^, IQR 3.62–7.87, age- and sex-adjusted, *p* = 0.002) and D-dimer levels (1572, IQR 1003–3859 vs. 1046 ng/dL, IQR 640–2025, age- and sex-adjusted, *p* = 0.010). Patients with VTE had the worst O_2_ saturation during hospitalization (88, IQR 83–91 vs. 92, IQR 88–94, age- and sex-adjusted, *p* = 0.002) and experienced significantly higher rates of NIVS (44% vs. 8%) and sedative therapy (50% vs. 26%). No statistical differences in mortality rate were found (41% vs. 27%); however, patients with VTE were more often transferred to the intensive care unit (ICU) (19% vs. 4%).

### 3.2. Multivariate Logistic Regression Model

Using a stepwise multivariate logistic regression model (Table 2), the presence of ECG abnormalities (OR 2.722, 95% confidence interval (CI) 1.039–7.133, *p* = 0.042), cough as symptom of presentation (OR 3.019, 95% CI 1.265–7.202, *p* = 0.013), neutrophil (OR 1.089, 95% CI 1.015–1.169, *p* = 0.018), CHA2DS2-VASC score > 3 (OR 3.404, 95% CI 1.362–8.513, *p* = 0.009) and the use of NISV (OR 15.530, 95%, CI 6.244–38.627, *p* < 0.001) were independently associated with VTE during hospitalization in SARS-CoV-2 patients.

**Figure 1:**
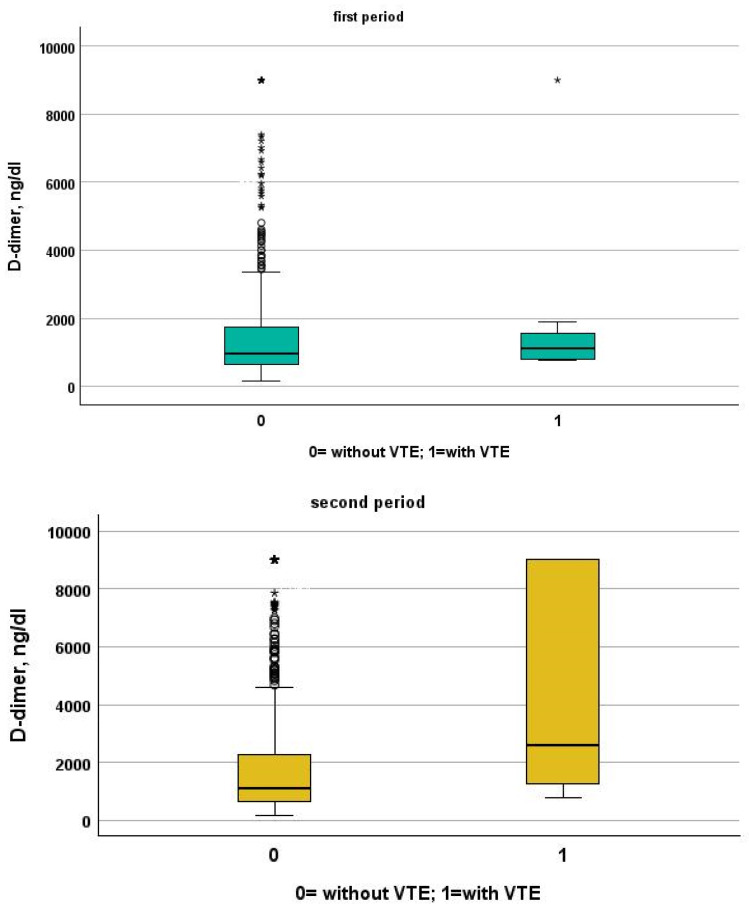
Comparison between D-dimer levels in patients with and without VTE in the first and second period, respectively.

**Figure 2:**
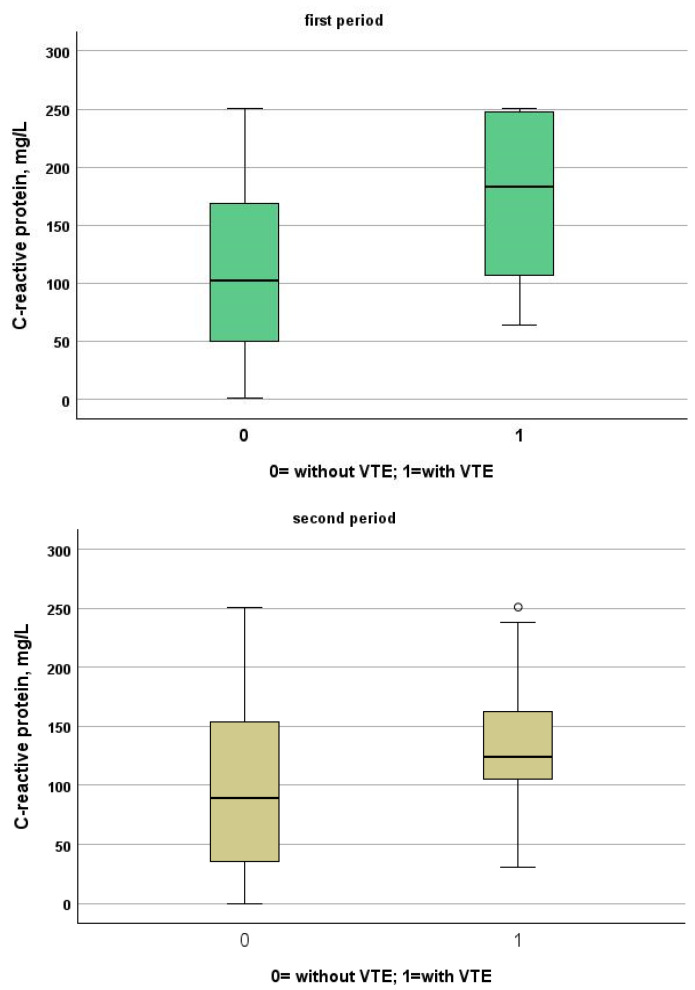
Comparison between C-reactive protein levels in patients with and without VTE in the first and second period, respectively.

**Figure 3:**
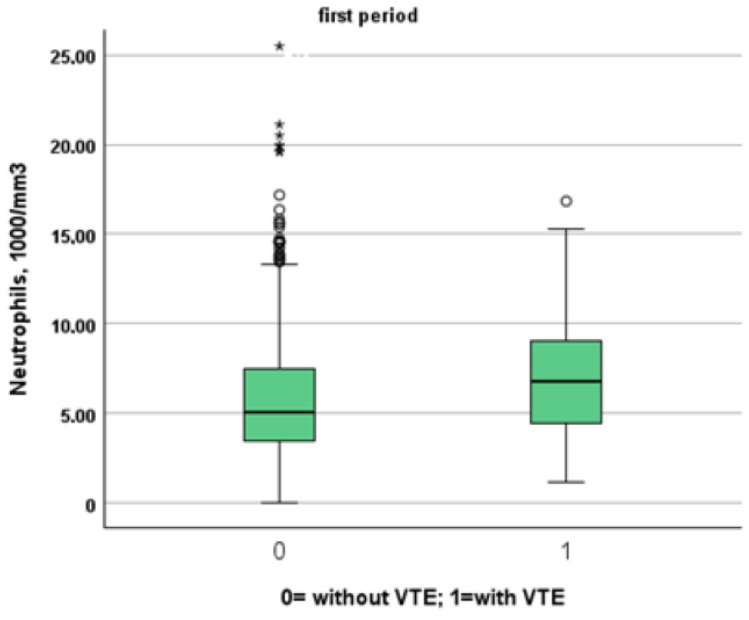

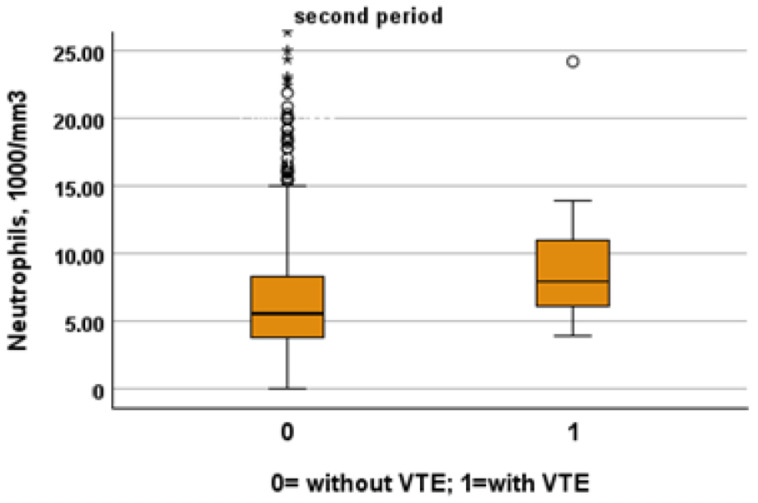
Comparison between neutrophil levels in patients with and without VTE in the first and second period, respectively.

**Figure 4:**
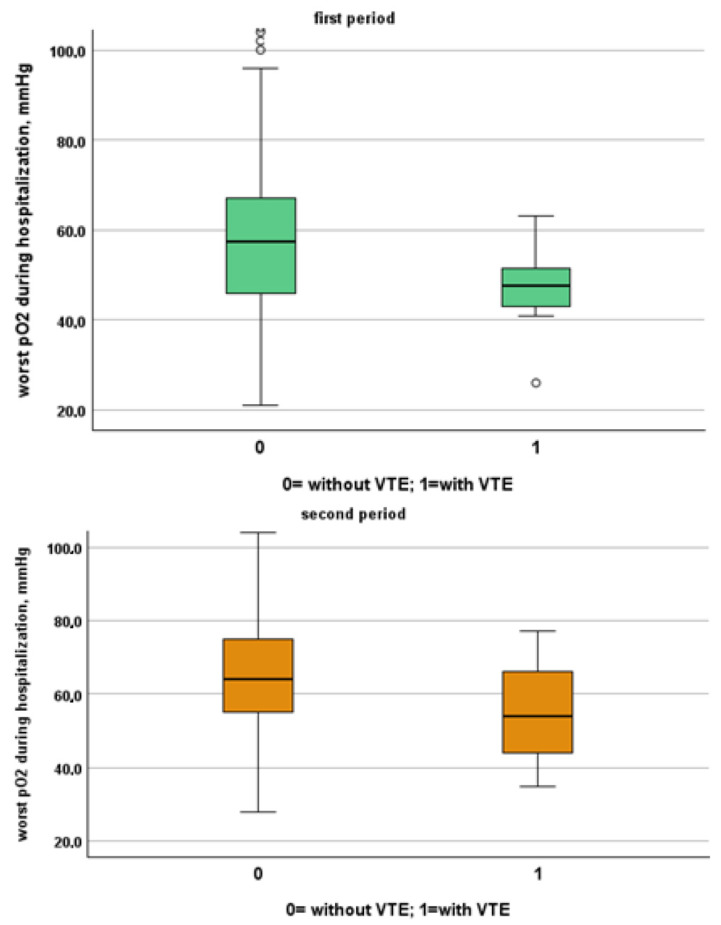
Comparison between worst O_2_ levels during hospitalization in patients with and without VTE in the first and second period, respectively.

Table 3 and Table 4 show a comparison between patients of the two study periods with and without VTE. Patients from the first period were younger than patients from the second period (patients without VTE: 72, IQR 61–81 vs. 80, IQR 68–88; *p* < 0.001, VTE: 66, IQR 54–77 vs. 85, IQR 80–88, *p* = 0.001). Despite this, NIVS remains an independent risk factor for VTE in the first (OR 30.297, 95% CI 7.344–124.990, *p* < 0.001) and in the second period (OR 9.825, 95% CI 2.136–45.181, *p* = 0.003). See Table 5 and Table 6 for more details. Table 7 shows the drugs used for the treatment of VTE and their mechanism of action. Finally in Figure 1, Figure 2, Figure 3 and Figure 4 we compare some key parameters during hospitalization in patients with and without VTE in the first and second period, respectively.

## 4. Discussion

In this retrospective study, we demonstrated that patients hospitalized for SARS-CoV-2 during the first and second periods who experienced NIVS during hospitalization, regardless of intensive care unit (ICU) transfer, exhibited a higher risk of VTE compared to patients who did experience NIVS. Patients from the descending phase of the first pandemic wave were younger and had fewer chronic comorbidities. Despite this, the risk of VTE remained higher in NIVS patients. NIVS was used for all patients suffering from complicated forms of SARS-CoV-2 infection. In this sense, the use of NIVS is an indicator of severe/complicated SARS-CoV-2 infection. Moreover, the causal effect of NIVS on VTE is linked to the severity of the SARS-CoV-2 infection.

The high incidence of VTE in critically ill patients with SARS-CoV-2 has been well established [16,22]; however, the incidence of VTE in patients receiving NIVS was only partially evaluated [23].

All hospitalized patients with an acute medical illness are at high VTE risk. Coagulopathy, uncontrolled inflammatory reaction and endothelial dysfunction are the main reasons for the development of thrombotic complications in SARS-CoV-2 [24]. These pathways are present at all the stages of the disease, but especially in patients requiring ICU and NIVS [18,19]. This was because patients admitted to ICUs and with an NIVS prescription have ICU-specific risk factors (sedation, immobilization and central venous catheters), and individual patient-related risk factors for VTE (obesity, age, history of personal or familial VTE, cancer, sepsis, respiratory or heart failure, stroke, trauma or recent surgery) [5,6,10]. Case series studies and case reports conducted in an ICU setting reported high VTE prevalence, particularly in patients with a severe SARS-CoV-2 infection [25,26,27,28]. It has been suggested that SARS-CoV-2 in severe forms of the disease induces an excessive inflammatory state via cytokine storm combined with endothelial injury and pulmonary vascular micro thrombosis. In line with this, it has been hypothesized that the origin of the increased D-dimer is intra-alveolar fibrin deposition in the context of severe acute lung injury [29]. Finally, hypertension, obesity, dyslipidemia and diabetes were highly incident among both groups of patients [30]. Our results are in line with previously published evidence that found a prevalence of between 26 and 77% of PE diagnoses in patients with SARS-CoV-2 and respiratory deterioration, depending on whether they were in the emergency room, a general ward or ICU [31].

Data from our study reflect routine, unmonitored medical practice involving a broad spectrum of patients with suspected or confirmed SARS-CoV-2 infection. They can, therefore, provide insights into the natural history of SARS-CoV-2 and assist in generating hypotheses. However, there are several limitations that need to be addressed. This is an observational study with a retrospective design, and no randomization to SARS-CoV-2 versus no SARS-CoV-2 patients. Thus, some non-measured variables have not been considered. We performed an extensive adjustment for several variables that may have impacted the incidence of VTE and the findings remained robust. Nevertheless, residual confounding factors may remain, as certain potential confounding variables may not have been available or may not have had the desired level of granularity. Furthermore, patients included in this study were admitted during a period of unprecedented burden of workload for the host institution, due to the large number of subjects with severe respiratory failure needing urgent admission. This circumstance required a significant and quick reorganization of all hospital activities [20]. In this setting, we cannot exclude the underdetection of VTE as a complication of COVID-19 during hospital stays. However, since all subjects included in the study underwent a chest CT before ward admission, the presence of VTE or pulmonary embolism upon admission was reliably detected. Finally, our study does not provide direct mechanistic insights into how NIVS may influence outcomes. Further research, including experimental and clinical studies, is needed to elucidate the underlying biological pathways and confirm causality.

## 5. Conclusions

In conclusion, the cumulative incidence of VTE is significantly higher in critically ill patients with a potential impact on the evolution of SARS-CoV-2. In our study, we highlight the role of uncontrolled inflammatory reactions and endothelial dysfunction as the main reasons for the development of thrombotic complications in SARS-CoV-2. In this context, patients undergoing NIVS represent a group at very high risk of developing VTE without a clear strategy regarding thromboprophylaxis. Our approach in patients with SARS-CoV-2 that receive NIVS is to initiate regular thromboprophylaxis together with active surveillance through the use of daily clinical and instrumental evaluations through compression ultrasound (CUS) or echo-color-Doppler evaluation. Future studies should evaluate whether thromboprophylaxis needs to be specifically recommended for patients with severe COVID-19 requiring NIVS.

## Figures and Tables

**Table 1:** Comparison of the main characteristics of COVID-19 presentation and outcomes between patients with venous thromboembolism (VTE) (*n* = 27) and without VTE (*n* = 1444).

	Patients without VTE *n* = 1444	Patients with VTE *n* = 27 (1.8%)	*p*	*p* Adjusted for Age and Sex
Demography and personal history				
Age, years	76 (63–84)	79 (66–88)	0.482	
Females, %	45	52	0.511	
Chronic illnesses, number	3 (1–5)	3 (2–5)	0.175	0.251
CHA2DS2Vasc Score	3 (1–4)	4 (2–5)	0.034	0.021
Diabetes, %	21	41	0.012	0.015
Obesity, %	11	26	0.017	0.013
Hypertension, %	59	74	0.114	0.140
Chronic heart disease, %	26	22	0.629	0.520
Dyslipidemia, %	17	19	0.839	0.837
Cancer, %	16	11	0.508	0.462
Arrhythmias, %	15	19	0.615	0.737
Vasculopathies, %	10	19	0.147	0.188
Dementia, %	18	22	0.566	0.783
Epilepsy, %	4	7	0.327	0.354
Drugs, number	4 (1–6)	4 (1–7)	0.892	0.782
Drugs AVKs/DOACs, %	13	15	0.788	0.913
Antiaggregants, %	30	33	0.757	0.847
Antiepileptics, %	7	19	0.026	0.041
Hypoglycemic, %	13	27	0.049	0.063
Insulin, %	7	19	0.015	0.023
Clinical presentation upon admission				
Symptom duration, days	7 (3–10)	7 (5–10)	0.692	0.523
Fever, %	81	81	0.983	0.896
Dyspnea, %	54	46	0.424	0.377
Cough, %	44	65	0.029	0.021
CT-ground glass, %	94	93	0.719	0.833
CT visual score, %	30 (20–50)	40 (25–60)	0.058	0.053
RT-PCR positive on admission, %	63	63	0.972	0.926
ECG on admission				
ECG abnormalities, %	52	74	0.022	0.028
Atrial fibrillation, %	12	15	0.702	0.794
QT interval, millisecond	384 (356–418)	398 (337–425)	0.956	0.955
QT correct, millisecond	449 (430–473)	444 (424–471	0.575	0.439
Arterial blood gas analysis on admission				
pH	7.44 (7.41–7.47)	7.46 (7.43–7.49)	0.322	0.246
HCO_3_^−^, mmol/L	25 (23–27)	26 (24–28)	0.072	0.059
pCO_2_, mmHg	36 (33–40)	38 (35–40)	0.166	0.175
pO_2_, mmHg	75 (63–95)	74 (62–87)	0.632	0.603
pO_2_/FiO_2_, mmHg	256 (147–349)	205 (145–311)	0.314	0.342
Blood tests on admission				
WBC, 1000/mm^3^	6.99 (5.12–9.55)	9.16 (6.62–12.50)	0.010	0.011
Neutrophils, 1000/mm^3^	5.32 (3.62–7.87)	7.65 (5.43–10.13)	0.002	0.002
Lymphocytes, 1000/mm^3^	0.92 (0.63–1.28)	0.82 (0.50–1.16)	0.144	0.148
C-reactive protein, mg/L	96 (44–162)	138 (105–220)	0.001	0.001
D-dimer, ng/dL	1046 (640–2025)	1572 (1003–3859)	0.020	0.010
D-dimer ≥ 9000 ng/dL, %	7	21	0.017	0.013
Creatinine, mg/dL	0.9 (0.7–1.2)	1.1 (0.8–1.3)	0.307	0.267
PCT, ng/mL	0.16 (0.07–0.48)	0.19 (0.13–0.45)	0.202	0.220
PT	1.21 (1.13–1.32)	1.18 (1.11–1.33)	0.504	0.549
INR ratio	1.21 (1.13–1.33)	1.22 (1.13–1.33)	0.922	0.848
aPTT ratio	0.97 (0.90–1.07)	0.93 (0.88–1.03)	0.082	0.093
Fibrinogen, mg/dL	596 (469–730)	612 (525–866)	0.236	0.189
Clinical course				
Worst O_2_ saturation during hospitalization, %	92 (88–94)	88 (83–91)	0.002	0.002
Worst pO_2_ during hospitalization, mmHg	60 (50–72)	51 (43–61)	0.002	0.002
Maximum oxygen flow, %	40 (28–75)	75 (36–95)	0.001	0.001
Max temperature during hospitalization, °C	37.6 (36.8–38.5)	38.2 (37.7–38.8)	0.024	0.010
Non-invasive ventilation support, %	8	44	<0.001	<0.001
Peripherial ischemia, %	0.3	0.0	0.784	/
Ictus, %	0.6	0.0	0.698	/
Sedation, %	26	50	0.007	0.010
Therapies				
Antivirals, %	43	67	0.016	0.012
Antibiotics %	92	100	0.127	/
Penicillins, %	81	96	0.046	0.075
Linezolid, %	5	33	<0.001	<0.001
Hydroxychloroquine, %	57	93	<0.001	0.002
Colchicine, %	7	19	0.022	0.021
Steroids, %	19	33	0.072	0.081
Tocilizumab, %	3	30	<0.001	<0.001
Enoxaparin, %	90	100	0.077	/
Enoxaparin dose,	6000 (4000–8000)	10000 (8000–16,000)	<0.001	<0.001
Fondaparinux dose, %	2.5 (2–5)	7.5 (7.5–7.5)	0.090	0.101
Outcomes				
Death, %	27	41	0.104	0.119
Intensive care unit, %	4	19	**<0.001**	**<0.001**

Data are shown as median and interquartile ranges or percentages. Crude comparisons were made with Mann–Whitney test or chi-square test, as appropriate. *p* adjusted for age and sex with ANCOVA (non-parametric of Quade) or binary logistic regression. *p* values < 0.05 are indicated in bold.

**Table 2:** Factors associated with the risk of VTE using a stepwise multivariate logistic regression analysis.

	Odds Ratio	95% Confidence Interval	*p*
Non-invasive ventilation support	15.530	6.244–38.627	<0.001
CHA2DS2Vasc Score > 3	3.404	1.362–8.513	0.009
Cough	3.019	1.265–7.202	0.013
Neutrophils, 1000/mm^3^	1.089	1.015–1.169	0.018
ECG abnormalities	2.722	1.039–7.133	0.042

Variables considered in the model: diabetes, CHA2DS2-VASC SCORE, obesity, ECG abnormalities on admission, cough, dyspnea, neutrophils, C-reactive protein, non-invasive ventilation support and CHA2DS2-VASC SCORE > 3, CHA2DS2-VASC SCORE > 4.

**Table 3:** Comparison of the main characteristics of COVID-19 presentation and outcomes between patients with and without venous thromboembolism hospitalized during the first period.

First Period 28 February–22 March 2020				
	Patients without VTE *n* = 766	Patients with VTE *n* = 12 (1.5%)	*p*	*p* Adjusted for Age and Sex
Age	72 (61–81)	66 (54–77)	0.168	
Females, %	40	25	0.294	
Diabetes, %	19	42	0.056	**0.042**
Obesity, %	12	42	**0.002**	**0.009**
CHA2DS2Vasc Score	2 (1–4)	3 (2–4)	0.556	**0.004**
Cough, %	52	83	**0.034**	0.080
Dyspnea, %	49	33	0.283	0.366
CT visual score, %	30 (20–50)	45 (28–63)	0.114	0.101
ECG abnormalities, %	46	58	0.382	0.175
Blood tests on admission				
WBC, 1000/mm^3^	6.52 (4.9–9.05)	8.06 (5.38–10.82)	0.251	0.206
Neutrophils, 1000/mm^3^	5.03 (3.45–7.45)	6.76 (4.26–9.55)	0.151	0.113
C-reactive protein, mg/L	102 (50–170)	184 (106–249)	**0.005**	**0.003**
CRP ≥ 250 mg/L, %	9	25	0.051	0.051
D-dimer, ng/dL	980 (636–1747)	1111 (804–1734)	0.389	0.141
D-dimer ≥ 9000 ng/dL, %	7	11	0.589	0.537
aPTT ratio	0.97 (0.90–1.05)	0.94 (0.85–1.04)	0.380	0.340
Clinical course				
Worst O_2_ saturation during hospitalization, %	91 (85–94)	88 (82–90)	**0.021**	**0.006**
Worst pO_2_ during hospitalization, mmHg	58 (46–67)	48 (43–53)	**0.030**	**0.024**
Maximum oxygen flow, %	44 (28–75)	85 (76–100)	**<0.001**	**<0.001**
Max temperature during hospitalization, °C	38.0 (37.1–38.7)	38.8 (38.4–39.0)	**0.004**	**0.008**
Non-invasive ventilation support, %	10	75	**<0.001**	**<0.001**
Sedation, %	28	67	**0.003**	**0.004**
Outcomes				
Death, %	28	33	0.688	0.314
Intensive care unit ICU, %	5	33	**<0.001**	**0.001**

Data are shown as median and interquartile ranges or percentages. Crude comparisons were made with Mann–Whitney test or chi-square test, as appropriate. *p* adjusted for age and gender with ANCOVA (non-parametric of Quade) or binary logistic regression. *p* values < 0.05 are indicated in bold.

**Table 4:** Comparison of the main characteristics of COVID-19 presentation and outcomes between patients with and without venous thromboembolism hospitalized during the second period.

	**Second Period** **23 March–10 June 2020**			
	**Patients without VTE** ***n*** **= 678**	**Patients with VTE** ***n*** **= 15 (2.2%)**	***p***	***p*** **Adjusted for Age and Sex**
Age	80 (68–88)	85 (80–88)	0.102	
Females, %	52	73	0.099	
Diabetes, %	22	40	0.099	0.096
Obesity, %	10	13	0.709	0.443
CHA2DS2Vasc Score	3 (2–5)	5 (4–6)	**0.022**	0.115
Cough, %	34	50	0.215	0.112
Dyspnea, %	54	46	0.424	0.378
CT visual score, %	30 (20–50)	33 (25–60)	0.290	0.274
ECG abnormalities, %,	59	87	**0.029**	0.089
Blood tests on admission				
WBC, 1000/mm^3^	7.46 (5.51–10.37)	10.02 (6.98–13.64)	**0.023**	**0.036**
Neutrophils, 1000/mm^3^	5.61 (3.77–8.39)	7.99 (6.14–11.81)	**0.007**	**0.012**
C-reactive protein, mg/L	89 (36–154)	124 (104–164)	**0.033**	**0.013**
CRP ≥ 250 mg/L, %	6	7	0.923	0.814
D-dimer, ng/dL	1101 (657–2260)	2621 (1233–9000)	**0.014**	**0.022**
D-dimer ≥ 9000 ng/dL, %	7	30	**0.007**	**0.008**
aPTT ratio	0.99 (0.90–1.08)	0.92 (0.88–1.01)	0.125	0.184
Clinical course				
Worst O_2_ saturation during hospitalization, %	93 (90–95)	90 (86–93)	**0.020**	**0.031**
Worst pO_2_ during hospitalization, mmHg	64 (55–75)	54 (43–67)	**0.016**	**0.014**
Maximum oxygen flow, %,	36 (28–75)	36 (27–85)	0.632	0.576
Max temperature during hospitalization,	37.3 (36.0–38.0)	37.8 (36.0–38.2)	0.343	0.148
Non-invasive ventilation support, %	6	20	**0.024**	**0.014**
Sedation, %	25	36	0.362	0.480
Outcome				
Death, %	25	47	0.060	0.125
Intensive care unit ICU, %	2	7	0.196	0.107

Data are shown as median and interquartile ranges or percentages. Crude comparisons were made with Mann–Whitney test or chi-square test, as appropriate. *p* adjusted for age and sex with ANCOVA (non-parametric of Quade) or binary logistic regression. *p* values < 0.05 are indicated in bold.

**Table 5:** Factors associated with the risk of VTE according to stepwise multivariate logistic regression analysis during the first period.

	Odds Ratio	95% Confidence Interval	*p*
Non-invasive ventilation support	30.297	7.344–124.990	<0.001
Dyspnea	0.159	0.039–0.647	0.010
C-reactive protein	1.010	1.001–1.020	0.027

Variables considered in the model: diabetes, CHA2DS2-VASC SCORE, obesity, ECG abnormalities on admission, cough, dyspnea, neutrophils, C-reactive protein, non-invasive ventilation support and CHA2DS2-VASC SCORE > 3, CHA2DS2-VASC SCORE > 4.

**Table 6:** Factors associated with the risk of VTE according to stepwise multivariate logistic regression analysis during the second period.

	**Odds Ratio**	**95% Confidence Interval**	***p***
Non-invasive ventilation support	9.825	2.136–45.181	0.003
CHA2DS2-VASC SCORE	1.568	1.084–2.267	0.017
Neutrophils, 1000/mm^3^	1.105	1.009–1.211	0.032

Variables considered in the model: diabetes, CHA2DS2-VASC SCORE, obesity, ECG abnormalities on admission, cough, dyspnea, neutrophils, C-reactive protein, non-invasive ventilation support and CHA2DS2-VASC SCORE > 3, CHA2DS2-VASC SCORE > 4.

**Table 7:** Drugs used in treatments of venous thromboembolism, their pharmacodynamics and mechanism of action.

Drugs	Pharmacodynamics	Mechanism of Action
Enoxaparin	This drug has an immediate onset of action. Enoxaparin increases thrombin time (TT) and activated partial thromboplastin time (aPTT). Administered at 1.5 mg/kg subcutaneously, enoxaparin led to a higher ratio of anti-Factor Xa to anti-Factor IIa activity. Enoxaparin administered at 1 mg/kg subcutaneously every 12 h led to aPTT values of 45 s or less in most patients.	Enoxaparin binds to antithrombin III, a serine protease inhibitor, forming a complex that irreversibly inactivates factor Xa, which is frequently used to monitor anticoagulation in the clinical setting. Following factor Xa inactivation, enoxaparin is released and binds to other anti-thrombin molecules. Factor IIa (thrombin) is directly inhibited by enoxaparin. Due to the cascade of effects resulting from enoxaparin binding, thrombin is unable to convert fibrinogen to fibrin and form a clot
Fondaparinux	Fondaparinux binds specifically to the natural anticoagulant factor, ATIII. Binding to ATIII potentiates the neutralizing action of ATIII on Factor Xa 300-fold. Neutralization of Factor Xa decreases the conversion of prothrombin to thrombin, which subsequently decreases the conversion of fibrinogen to fibrin (loose meshwork). The decrease in thrombin also decreases the activation of Factor XIII, which decreases the conversion of fibrin in its loose meshwork form to its stabilized meshwork form. Disruption of the coagulation cascade effectively decreases the formation of blood clots. Fondaparinux does not inactivate thrombin (activated Factor II). According to the manufacturer, fondaparinux has no known effect on platelet function.	The antithrombotic activity of fondaparinux is the result of the ATIII-mediated selective inhibition of Factor Xa. By selectively binding to ATIII, fondaparinux potentiates (about 300 times) the neutralization of Factor Xa by ATIII. Neutralization of Factor Xa interrupts the blood coagulation cascade and thus inhibits thrombin formation and thrombus development.

## Data Availability

The raw data supporting the conclusions of this article will be made available by the authors upon reasonable request, in accordance with the current legislation.
